# Supplementary material for: The first complete hand-rearing of two neonatal finless porpoises
Source: PLoS One. 2025 Jan 30;20(1):e0288891. doi: 10.1371/journal.pone.0288891 (PMC11781668; doi:10.1371/journal.pone.0288891)
Supplement: Attachment — Submitted filename: PONE-D-23-20946-Reviewer1.docx [file pone.0288891.s002.docx]

**General comment:**

This paper firstly reported two cases of hand-rearing of two Eastern Asian finless porpoise babies and provided valuable technical information and data of this process, which is very helpful and informative to other researchers and rescuers when facing the similar situation of hand-rearing orphans of small cetacean species. I suggest accepting this paper with minor improvements. Moreover, I suggest the authors to further improve the language by native speakers for a better quality of this paper.

**Detailed suggestions:**

1. Line 24: the authors use “daily number of lactations”. Is it more proper to use “frequency of lactations”?
2. Line 33-38: Reorganize the last three sentences of the abstract to make a conclusion of this work.
3. Line 46: suggest changing “large number” to “high frequency”.
4. Line 60-62: Is there any information to compare the pros and cons of the two methods of hand-nursing the small cetacean babies?
5. Line159: What is the frequency of blood sampling and how many samples are collected for different analysis?
6. Line204-208: I suggest the authors to calculate the “Amount of each lactation” by body weight, which I think is more helpful for other researchers when they handle other species of small cetacean with different body weight.
8. Line236: In table 2: are the values of week and month data the mean value or a one test value? If they are mean values, the authors should provide the SD value for each data.
9. Line239-244: same suggestions to figure 3. This is also even more important for other cetacean species.
10. Line270-277: The sample number should be presented. Did the AA of two babies change during the study period? If it changes with growth, what is the trend of the changes?
11. Line304-308: Did the authors tried the buttle-feeding method? Why do you think tube-feeding is better than bottle-feeding?
12. Line322-323: This information should be presented in the result section with figures.
